# Identification of an Interferon-Stimulated Long Noncoding RNA (LncRNA ISR) Involved in Regulation of Influenza A Virus Replication

**DOI:** 10.3390/ijms20205118

**Published:** 2019-10-16

**Authors:** Qidong Pan, Zhonghui Zhao, Yuan Liao, Shih-Hsin Chiu, Song Wang, Biao Chen, Na Chen, Yuhai Chen, Ji-Long Chen

**Affiliations:** 1 Key Laboratory of Fujian-Taiwan Animal Pathogen Biology, College of Animal Sciences, Fujian Agriculture and Forestry University, Fuzhou 350002, China; 13665015716@163.com (Q.P.); liaoyuan919@163.com (Y.L.); shchiu802@163.com (S.-H.C.); wscookie@163.com (S.W.); a747110207@163.com (N.C.); 2 CAS Key Laboratory of Pathogenic Microbiology and Immunology, Institute of Microbiology, Chinese Academy of Sciences, Beijing 100101, China; zhaozhong.hui@163.com (Z.Z.); chenbiaozhuifeng@163.com (B.C.); chenyuhai@im.ac.cn (Y.C.)

**Keywords:** Influenza A virus, long noncoding RNAs, viral replication, interferon, innate immunity

## Abstract

Long noncoding RNAs (lncRNAs) are involved in a diversity of biological processes. It is known that differential expression of thousands of lncRNAs occurs in host during influenza A virus (IAV) infection. However, only few of them have been well characterized. Here, we identified a lncRNA, named as interferon (IFN)-stimulated lncRNA (ISR), which can be significantly upregulated in response to IAV infection in a mouse model. A sequence alignment revealed that lncRNA ISR is present in mice and human beings, and indeed, we found that it was expressed in several human and mouse cell lines and tissues. Silencing lncRNA ISR in A549 cells resulted in a significant increase in IAV replication, whereas ectopic expression of lncRNA ISR reduced the viral replication. Interestingly, interferon-β (IFN-β) treatment was able to induce lncRNA ISR expression, and induction of lncRNA ISR by viral infection was nearly abolished in host deficient of IFNAR1, a type I IFN receptor. Furthermore, the level of IAV-induced lncRNA ISR expression was decreased either in retinoic acid-inducible gene I (*RIG-I*) knockout A549 cells and mice or by nuclear factor κ-light-chain-enhancer of activated B cells (NF-κB) inhibitor treatment. Together, these data elucidate that lncRNA ISR is regulated by *RIG-I*-dependent signaling that governs IFN-β production during IAV infection, and has an inhibitory capacity in viral replication.

## 1. Introduction

Influenza A virus (IAV) is an RNA virus belonging to the *Orthomyxoviridae* family, which contains eight segments of negative-sense single-stranded RNA, and it can give rise to severe respiratory diseases in humans and animals [1,2]. IAV infection can trigger host innate immune responses through engagement of pathogen recognition receptors (PRRs) such as retinoic acid-inducible gene I (*RIG-I*), melanoma differentiation-associated gene 5 (*MDA5*), Toll-like receptor 3 (TLR3), and Toll-like receptor 7 (TLR7) that associate with signaling to activate transcription factors such as interferon regulatory factors (IRFs) and nuclear factor κ-light-chain-enhancer of activated B cells (NF-κB), leading to the expression of various cytokines, including type I and III interferons (IFNs) [3,4,5,6]. These IFNs bind to their receptors to activate molecules downstream Janus kinase-signal transducer and activator of transcription (JAK-STAT) signaling that consequently initiates the transcription of interferon-stimulated genes (ISGs), including noncoding transcripts, which exert a board spectrum of antiviral effects [7,8,9,10,11,12,13,14,15,16,17]. 

Long noncoding RNAs (lncRNAs) are transcripts longer than 200 nucleotides in length that have no protein-coding potential or encode micropeptides, which are distinguished from short noncoding RNAs, such as microRNAs, small interfering RNAs, and piwi-interacting RNAs [18,19,20]. LncRNAs are involved in a wide variety of biological processes through diverse epigenetic modification for gene expression, mRNA stabilization, protein-protein and protein-RNA interaction, and molecule orientation either directly or indirectly [21]. Recently, host lncRNAs have been demonstrated to restrict the infection of diverse viruses, including IAV. During the course of viral infection, lncRNAs take part in modulating viral gene expression, viral RNA stability, cellular metabolism, and virus assembly and release. Importantly, it has been shown that host lncRNAs are involved in regulating innate immunity against viral infection [22].

First, previous studies reveal that lncRNAs play an important role in the regulation of PRRs. For example, lnczc3h7a can bind to tripartite motif protein 25 (TRIM25) for facilitating TRIM25-mediated K63-linked ubiquitination of *RIG-I*, thereby enhancing innate immune responses to viral infection [23]. Second, lncRNAs are associated with the production of IFNs and inflammatory cytokines. The induction of MIR155HG-derived lncRNA-155 by IAV infection causes increased production of IFN-β [22]. Furthermore, the IAV-induced lncRNA NEAT1 regulates interleukin-8 (IL-8) expression via directing splicing factor proline/glutamine-rich (SFPQ), a transcription repressor of IL-8, from the IL-8 promoter to nuclear body paraspeckles [24]. Third, lncRNAs can regulate the expression of some ISGs. For instance, the IAV-induced lnc-ISG20 enhances ISG20 translation and regulates viral replication [7]. On the other hand, expression of some lncRNAs may be favorable for viral infection and replication. IAV-induced lncRNA-PAAN and TSPOAP1-AS1 promote viral replication through regulating viral RNA-dependent RNA polymerase and IFN-β transcription [25,26]. LncRNA NRAV suppresses expression of ISGs by modulating histone modification in response to infection with IAV [27]. So far, accumulating evidences have revealed that the distinct expression of thousands of host lncRNAs can be regulated by IAV infection and might play integral roles in the virus-host interaction. However, the functions of many lncRNAs are still largely unknown.

Here, we identified a novel lncRNA whose expression was significantly increased during IAV infection both *in vivo* and *in vitro*. It was IFN-β inducible and thus named Interferon-stimulated lncRNA (lncRNA ISR). We found that lncRNA ISR was capable of suppressing IAV replication, and its expression was modulated through *RIG-I* and NF-κB-dependent pathway in response to IAV. Based on these observations, this study clarifies that IAV-induced lncRNA ISR participates in host antiviral defense.

## 2. Results

### 2.1. IAV Infection Markedly Induces Mouse lncrna ISR Expression In Vivo and In Vitro

**Figure 1:**
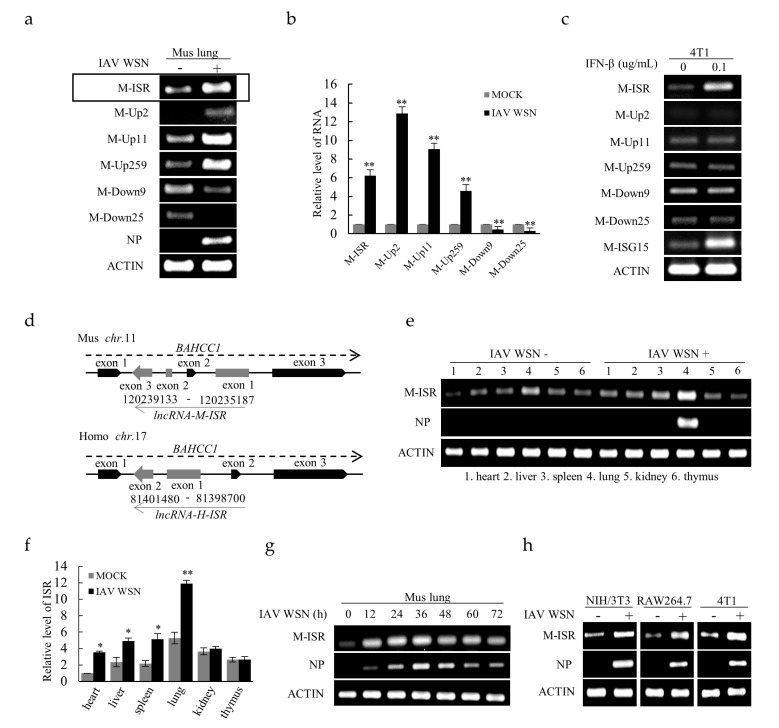
Induction of long noncoding RNA (lncRNA) expression in response to influenza A virus (IAV) infection. (**a**,**b**) Differential expression of six selected lncRNAs in mouse lung infected with or without influenza A/WSN/1933 (IAV WSN) was examined by reverse transcriptase-polymerase chain reaction (RT-PCR) and quantitative real time-polymerase chain reaction (qRT-PCR). Interferon-stimulated lncRNA (lncRNA ISR) is indicated by a rectangle. Data are represented as mean ± S.D. ** *p* < 0.01; (**c**) 4T1 cells were treated with interferon-β (IFN-β) for 3 h. The expression of selected lncRNAs were determined by RT-PCR; (**d**) Shown is a diagrammatic drawing of the genomic location of lncRNA ISR gene on mouse and human genomes; (**e**,**f**) The levels of lncRNA ISR expression in the organs of mice infected with or without IAV WSN for 24 h were measured by RT-PCR (**e**) and qRT-PCR (**f**). Lane 1–6: heart, liver, spleen, lung, kidney, and thymus. RT-PCR for detecting viral nucleoprotein (NP) was performed to indicate the virus infection. Data are represented as mean ± S.D. * *p* < 0.05; ** *p* < 0.01; (**g**) C57 black 6 (*C57BL/6*) mice were infected intranasally with 5 × 10^4^ pfu of IAV WSN virus for indicated time. The lungs were collected and subjected to RT-PCR; (**h**) The levels of lncRNA ISR in indicated mouse cell lines infected with or without IAV WSN (MOI = 0.8) for 16 h were examined by RT-PCR.

To explore the expression profile of lncRNAs in response to IAV infection, we utilized lncRNA microarrays to determine altered lncRNA expression in C57 black 6 (*C57BL/6)* mice infected with or without influenza A/WSN/1933 (WSN) virus. The analysis data (GEO: GSE80011) have been shown in our previous work [22]. Numerous upregulated lncRNAs and downregulated lncRNAs were observed in the lung homogenates of IAV-infected mice in comparison with non-infected controls. Based on these data, six lncRNAs whose expression was significantly changed were selected for further validation by reverse transcriptase-polymerase chain reaction (RT-PCR) and quantitative real time-polymerase chain reaction (qRT-PCR) (Figure 1a,b). Of these, lncRNA ISR (MN397202), Up2 (AK149792), Up11 (AK152734) and Up259 (FR239089) were markedly increased upon IAV infection. Alignment of sequences utilizing GenBank database showed that lncRNA ISR had highly homologous sequences between mice and human beings (Figure S1). Moreover, we found that only lncRNA ISR was induced by IFN-β treatment (Figure 1c). Therefore, lncRNA ISR was selected for further investigation. The mouse lncRNA ISR gene is located on chromosome 11, while the human lncRNA ISR gene is located on chromosome 17 (Figure 1d).

Next, expression of lncRNA ISR was examined in several mouse tissues and cell lines infected with or without IAV. We found that lncRNA ISR was detectable in heart, liver, spleen, lung, kidney and thymus, and significantly increased in heart, liver, spleen, and lung after the IAV infection (Figure 1e,f and Figure S2a). Interestingly, the highest level of lncRNA ISR was observed in lung and it was most significantly induced in lung infected with IAV as compared with other organs by RT-PCR and qRT-PCR analysis (Figure 1e,f). Furthermore, a time course study revealed that the increased expression of lncRNA ISR reached the peak about 24–36 h post infection (hpi) in the lung of IAV-infected mice (Figure 1g). Additionally, the increased expression of lncRNA ISR was found in mouse NIH/3T3 cells, RAW264.7 cells and 4T1 cells upon IAV infection (Figure 1h and Figure S2b). These results indicate that IAV infection can affect the expression of numerous lncRNAs, including lncRNA ISR.

### 2.2. Human lncRNA ISR Can be Induced by Several Virus Infections

**Figure 2:**
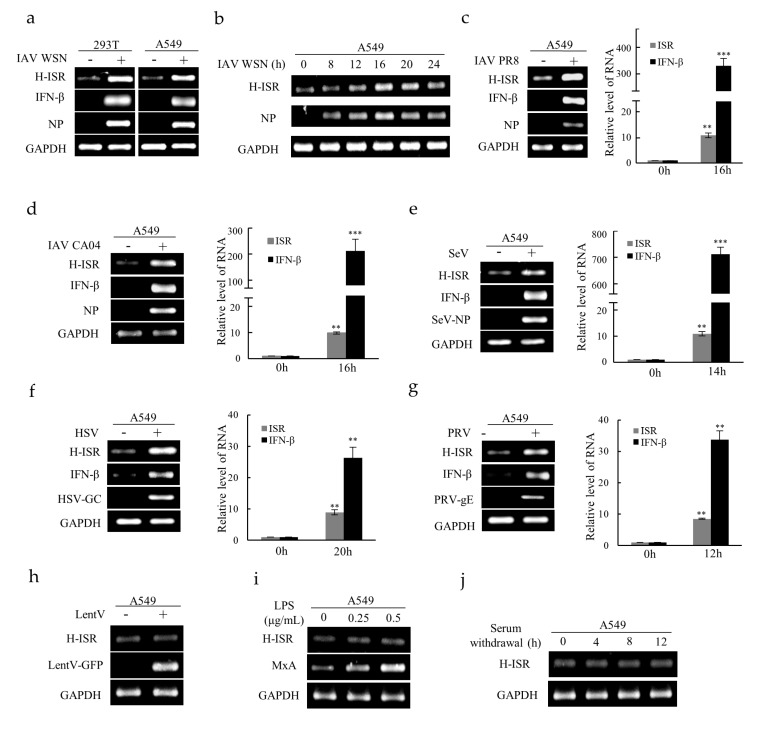
Human lncRNA ISR is induced by several virus infections. (**a**) The lncRNA ISR and IFN-β expression levels in indicated human cell lines infected with or without IAV WSN (MOI = 0.8) for 16 h was examined by RT-PCR; (**b**) A549 cells were infected with IAV WSN (MOI = 0.4) for indicated times. RT-PCR was performed to determine lncRNA ISR expression; (c–g) The lncRNA ISR and IFN-β expression levels were examined in A549 cells infected with IAV PR8 (**c**), IAV CA04 (**d**), HSV (**e**), SeV (**f**), PRV (**g**) by RT-PCR and qRT-PCR. Data are represented as mean ± S.D. ** *p* < 0.01; *** *p* < 0.001. A549 cells were transducted with pseudovirus (LentV) prepared by lentivirus expression system (**h**), or incubated with lipopolysaccharide (LPS) for 8 h (**i**), or cultured in serum-free media for indicated time (**j**). The expression of lncRNA ISR were determined by RT-PCR.

Several viruses were used to further determine whether lncRNA ISR could be induced after viral infection in human cells. In particular, the H-lncRNA ISR expression was dramatically upregulated in human 293T cells and A549 cells after IAV WSN infection (Figure 2a). Time-points experiment indicated that the H-lncRNA ISR expression reached the peak about 16 hpi in A549 cells (Figure 2b). Moreover, H-lncRNA ISR was remarkably upregulated by infections with other influenza virus subtypes, such as A/Puerto Rico/8/1934 (IAV PR8) and A/California/04/2009 (IAV CA04) (Figure 2c,d). Surprisingly, the increase in H-lncRNA ISR expression can be caused by several other viruses, including a negative ssRNA virus Sendai virus (SeV) (Figure 2e), a DNA virus herpes simplex virus (HSV) (Figure 2f), and a DNA virus Pseudorabies virus (PRV) (Figure 2g). Furthermore, expression of IFN-β was also upregulated after infection with these viruses (Figure 2a,c–g). In contrast, lncRNA ISR levels were not affected by pseudovirus transduction, lipopolysaccharide (LPS) treatment, or serum withdrawal (Figure 2h–j). Together, these results suggest that the upregulation of lncRNA ISR is related to viral infection.

### 2.3. LncRNA ISR Suppresses IAV Replication

**Figure 3:**
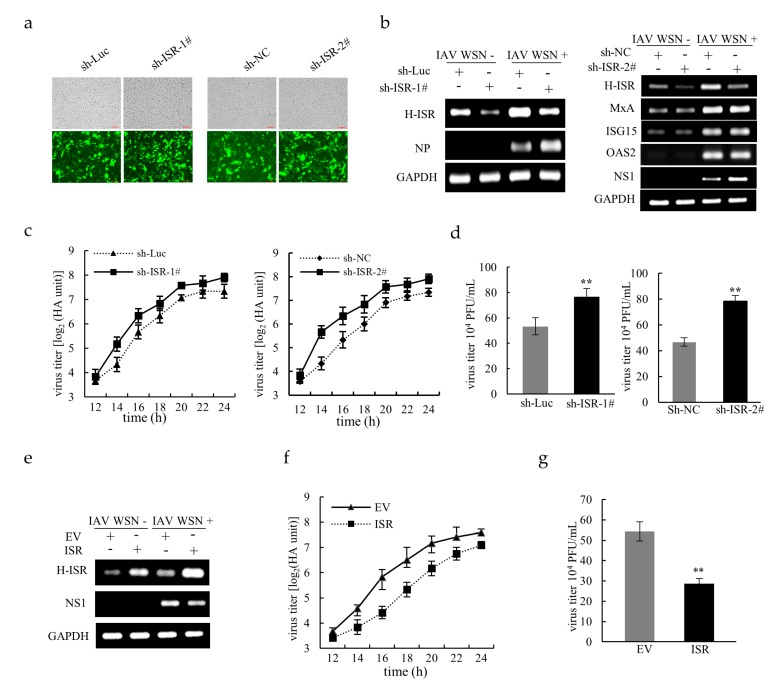
LncRNA ISR suppresses IAV replication. (**a**) Optical and their corresponding fluorescence images of A549 cells stably expressing pSIH-H1-GFP vectors targeting lncRNA ISR or luciferase control (Luc) or scramble nucleotide sequences (NC) (100 μm); (**b**) The A549 cells expressing two different sequences of lncRNA ISR-silencing shRNAs (sh-ISR-1# and sh-ISR-2#) or sh-Luc or sh-NC were infected with or without IAV WSN [MOI = 0.8 (left) MOI = 0.4 (right)] for 16 h. After infection, total RNA was extracted for RT-PCR to detect lncRNA ISR or ISGs (MxA, *ISG15* and OAS2) expression. RT-PCR for detecting viral NP or NS1 was performed to indicate the extent of viral replication. The cell culture supernatants were harvested at the indicated times for hemagglutination assay (**c**) and at 14 hpi for plaque assay (**d**) to measure virus titers. Data are represented as mean ± S.D. ** *p* < 0.01; (**e**) The A549 cells carrying either lncRNA ISR-expressing plasmid or EV were infected with or without IAV WSN (MOI = 0.4) for 16 h. After infection, total RNA was extracted for RT-PCR to detect lncRNA ISR expression. The cell culture supernatants were harvested at the indicated times for hemagglutination assay (**f**) and at 16 hpi for plaque assay (**g**) to measure virus titers. Data are represented as mean ± S.D. ** *p* < 0.01.

To ascertain whether lncRNA ISR is involved in regulating IAV replication, we knocked down and overexpressed lncRNA ISR in A549 cells through RNA interference and ectopic expression, respectively, followed by IAV infection. Green fluorescent protein (GFP) expression of transfected cells was confirmed over 80%, indicating a high transduction efficiency (Figure 3a). As shown in Figure 3b, silencing lncRNA ISR in A549 cells resulted in an increase in viral nucleoprotein (NP) or non-structural protein 1 (NS1) expression as compared with that in control cells expressing shRNA targeting luciferase (sh-Luc) or scrambled nucleotide sequences (sh-NC). However, knockdown of lncRNA ISR had little effect on the expression of several ISGs, including myxovirus resistance protein A (MxA), Interferon-stimulated gene 15 (*ISG15*) and human 2′-5′-oligoadenylate synthetase 2 (OAS2) (Figure 3b). Consistently, the virus titers measured by either hemagglutination assay (HA) or plaque assays were significantly increased in lncRNA ISR-knockdown cells compared to sh-Luc or sh-NC control (Figure 3c,d). Conversely, both of significant decreases in the NS1 mRNA level and the virus titers were observed in cells overexpressing lncRNA ISR as compared to those in cells transfected with empty vector (EV) control (Figure 3e–g). Additionally, it appeared that lncRNA ISR had the most significant effect on the IAV replication in 14–18 h post infection (Figure 3c,f). Altogether, these observations indicate that lncRNA ISR has the capability to suppress IAV replication.

### 2.4. RIG-I-Dependent Signaling Regulates IAV-Induced lncRNA ISR Expression

**Figure 4:**
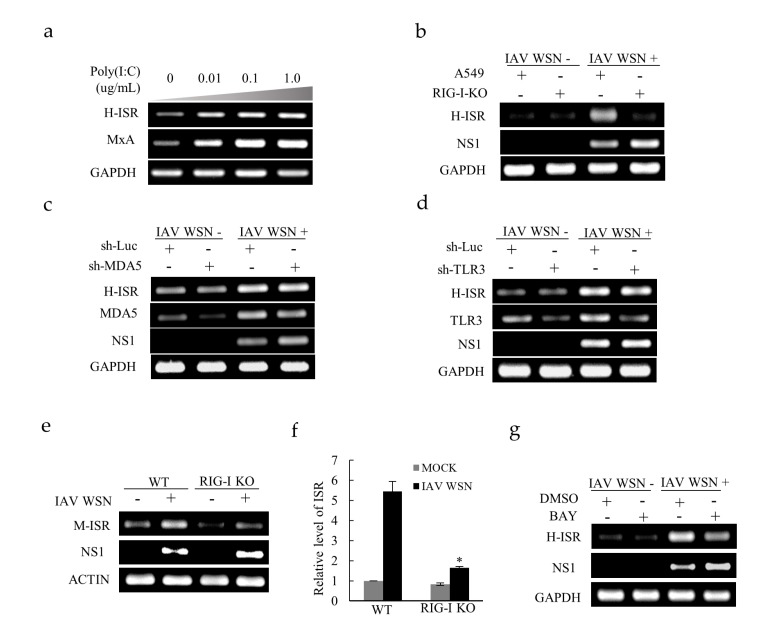
IAV-induced lncRNA ISR expression is regulated by *RIG-I*-dependent signaling. (**a**) A549 cells were treated with poly (I:C) at indicated concentrations for 4 h. RT-PCR was performed to determine lncRNA ISR expression; (**b**) A549 WT and A549 *RIG-I*-knockout (KO) cells were infected with or without IAV WSN (MOI = 0.8). At 16 hpi, total RNA was extracted for RT-PCR to detect lncRNA ISR expression; (**c**) The expression levels of lncRNA ISR in *MDA5* knockdown and sh-Luc control cells infected with or without IAV WSN (MOI = 0.8) were determined by RT-PCR; (**d**) The expression levels of lncRNA ISR in TLR3 knockdown and sh-Luc control cells infected with or without IAV WSN (MOI = 0.8) were determined by RT-PCR; (**e**,**f**) *C57BL/6* WT and *RIG-I*-KO mice were infected intranasally with or without 5 × 10^4^ pfu of IAV WSN virus (*n* = 8 mice/group). At 24 hpi, the lungs were collected and subjected to RT-PCR (**e**) and qRT-PCR (**f**) to detect lncRNA ISR expression. Data are represented as mean ± S.D. * *p* < 0.05; (**g**) A549 cells were treated with 8 μM NF-κB inhibitor BAY 11-7082 (BAY), followed by infection with/without IAV WSN (MOI = 0.8). At 16 hpi, total RNA was analyzed by RT-PCR to determine lncRNA ISR expression.

Poly (I:C), the viral mimetic polyinosinic, is a generally adopted tool to study the infection of RNA viruses. As expected, the lncRNA ISR expression increased in A549 cells after treatment with Poly (I:C) (Figure 4a). PRRs are host sensors that play an indispensible role in recognizing a variety of invading pathogens, also termed pathogen-associated molecular patterns (PAMPs), which initiate host innate immune responses including induction of molecules with antiviral functions, such as ISGs [28]. To determine how IAV infection induces lncRNA ISR expression, we assessed the effect of lacking *RIG-I* on lncRNA ISR expression in A549 cells by performing gene knockout. After viral infection, it was obvious that knockout of *RIG-I* reduced lncRNA ISR expression (Figure 4b). However, silence of *MDA5* and TLR3 had no significant effect on the expression of lncRNA ISR (Figure 4c,d). Furthermore, *RIG-I* knockout (KO) mouse model was employed and showed that the level of lncRNA ISR is significantly decreased in *RIG-I* KO lung compared to the wild type after IAV infection (Figure 4e,f). Thus, these results indicate that *RIG-I* contributes to lncRNA ISR expression upon IAV infection.

Previously, *RIG-I* has been identified to activate transcription factor NF-κB, leading to the production of IFN-β and subsequent molecules [29,30]. Since our study revealed that the induction of lncRNA ISR required *RIG-I* recognition, we carried out further experiments to address whether NF-κB took part in the downstream *RIG-I* signaling that mediates lncRNA ISR induction. As shown in Figure 4g, abolishment of NF-κB activity using its inhibitor BAY 11-7082 resulted in reduced level of lncRNA ISR expression in A549 cells, suggesting that NF-κB is involved in IAV-induced lncRNA ISR expression. These findings suggest that IAV infection upregulates lncRNA ISR through *RIG-I*-dependent signaling pathway involving NF-κB.

### 2.5. LncRNA ISR is Identified as an Interferon-Stimulated Gene

**Figure 5:**
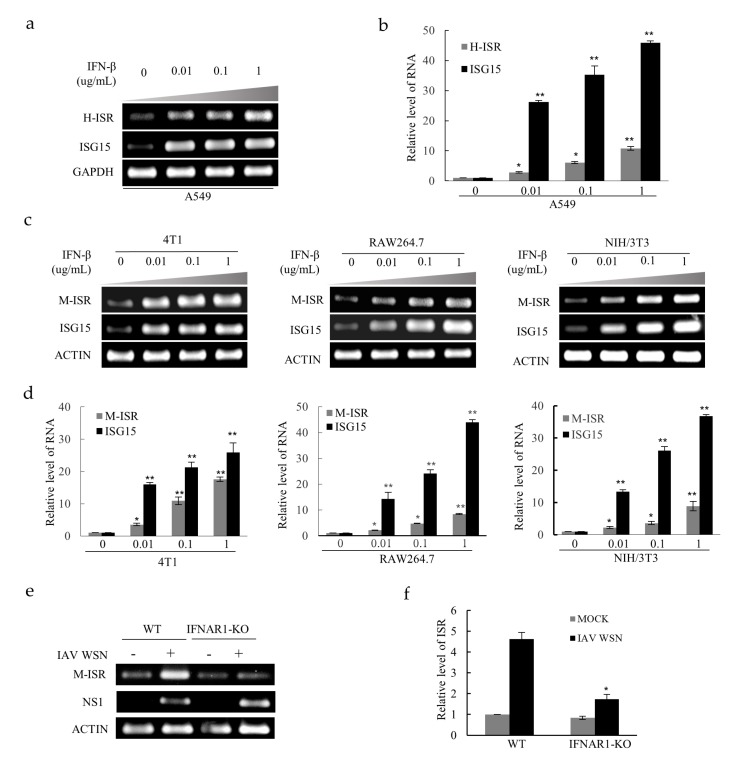
LncRNA ISR expression is induced by IFN-β. (**a**,**b**) A549 cells were treated with IFN-β at the indicated concentrations for 3 h. After treatment, total RNA was extracted for RT-PCR (**a**) and qRT-PCR (**b**) to detect lncRNA ISR expression. Data are represented as mean ± S.D. * *p* < 0.05; ** *p* < 0.01; (**c**,**d**) Mouse cells (4T1 cells, RAW264.7 cells, NIH/3T3 cells) were treated with IFN-β at indicated concentrations for 3 h. RT-PCR (**c**) and qRT-PCR (**d**) was performed to determine the lncRNA ISR expression. Data are represented as mean ± S.D. * *p* < 0.05; ** *p* < 0.01; (**e**,**f**) *C57BL/6* WT and IFNAR1-KO mice were infected with or without 5 × 10^4^ pfu of IAV WSN virus (*n* = 10 mice/group). At 16 hpi, total RNA was extracted for RT-PCR (**e**) and qRT-PCR (**f**) to detect lncRNA ISR expression. Data are represented as mean ± S.D. * *p* < 0.05.

ISGs have been widely reported that they are highly effective in controlling pathogens, and their expression is induced by IFN-mediated signaling. Based on the result that lncRNA ISR exerts an inhibitory effect on IAV replication as demonstrated above, we assumed that lncRNA ISR might be an ISG. To test this hypothesis, A549 cells were treated with IFN-β at indicated concentrations as shown in Figure 5a. RT-PCR and qRT-PCR analysis showed that expression level of lncRNA ISR was markedly increased by IFN-β treatment in a concentration-dependent manner (Figure 5a,b), suggesting that the production of lncRNA ISR is regulated by type I IFN-activated signaling. As a treatment control, IFN-β pronouncedly increased the expression of *ISG15*, a known ISG. Moreover, these results were also confirmed in several IFN-β-treated mouse cell lines such as 4T1 cells, RAW264.7 cells and NIH/3T3 cells (Figure 5c,d). In addition, we used a knockout (KO) mouse model deficient for interferon alpha/beta receptor 1 (IFNAR1) to confirm the role of type I IFN-activated signaling in regulation of lncRNA ISR expression. Indeed, levels of lncRNA ISR were significantly reduced in lung of IFNAR1-KO mice as compared with wild-type controls after IAV infection (Figure 5e,f). Together, these results indicate that lncRNA ISR may function as an ISG.

## 3. Discussion

An increasing number of studies reveal the presence of lncRNAs with regulatory roles in antiviral immunity. IFNs function as critical molecules to mediate the expression of numerous antiviral effectors, such as ISGs. Recently, it has been shown that production of lncRNAs can be regulated by IFNs [31,32]. These lncRNAs might be involved in regulation of IFN-mediated signaling responding to viral infection. For example, lncRNA-IFI6 induced by IFN-α is able to increase HCV replication via modulating function of ISG IFI6 promoter and histone modification [33]. IFN-α2 or -λ-induced lncBST2/BISPR, which is increased in the liver of patients infected with HCV, positively regulates ISG BST2 [34].

Nowadays, more and more reports have characterized the regulatory effects of IAV-induced lncRNAs on viral infection or host antiviral defense. Several such lncRNAs, including lnc-ISG20 and PSMB8-AS1, have been reported to be regulated by IFN signaling [7,8]. We herein treated A549 cells with IFN-β and also found that lncRNA ISR expression was upregulated by IFN-β treatment. In contrast, abolishment of the receptors specific to type I IFNs reduced the level of lncRNA ISR. We suggest that lncRNA ISR is an ISG whose expression is regulated by IFNs-activated signaling.

In this study, we elucidated that lncRNA ISR is able to suppress IAV replication measured by either HA assay, plaque assay or examination of viral NP and NS1 expression. However, data from HA assay showed more significant effects on IAV replication by altering lncRNA ISR expression than those obtained from plaque assay. Such difference might be due to plaque formation only by live viruses, while HA assay might detect viral components derived from live and dead viruses. Moreover, we observed that lncRNA ISR is not involved in regulating the expression of several critical ISGs. The molecular mechanism underlying the function of lncRNA ISR still needs to be determined. In addition, our data reveal that in the virus infected cells, increased expression of lncRNA ISR is regulated through *RIG-I*-dependent signaling pathway involving NF-κB. Taken together, these observations suggest that upregulation of lncRNA ISR is a host antiviral response. However, it remains to be addressed whether IRF3 and IRF7 are implicated in regulating lncRNA ISR expression downstream of *RIG-I*-dependent signaling. Moreover, we noticed that the decreased levels of lncRNA ISR expression were different between *RIG-I*-KO cells and the lungs of *RIG-I*-KO mice after IAV infection, and IFNAR1-KO mice showed complete suppression of lncRNA ISR expression. That is probably because of the complexity of the lung homogenate composition or the lncRNA ISR distribution in different types of lung cells. In addition, these results suggest that there are likely other pathways besides *RIG-I*/MAVS involved in triggering type I IFN production that induces lncRNA ISR in mice. Moreover, we found that lncRNA ISR is distributed in various tissues. The increased expression of lncRNA ISR in these tissues such as liver and spleen is likely induced by IFN-β after viral infection. Therefore, it is necessary to determine the associations between the distribution of lncRNA ISR in different tissues and its biological functions, especially antiviral immunity.

## 4. Materials and Methods

### 4.1. Viruses and Cells

Influenza A viruses, including A/WSN/1933(H1N1) (IAV WSN), A/Puerto Rico/8/1934 (IAV PR8), A/California/04/2009 (IAV CA04), were prepared as previously described [27]. Sendai virus (SeV) were propagated in specific pathogen free (SPF) embryonated chicken eggs, as previously described [27]. Pseudorabies virus (PRV) strain Min-A was propagated in Madin–Darby canine kidney cells (MDCK) cells, as previously described [35,36]. Herpes simplex virus 1 (HSV-1) was propagated in Vero cells as previously described [27]. For viral infection, cells were washed with phosphate-buffered saline (PBS) and infected with the indicated MOI of viruses in Dulbecco’s Modified Eagle’s Medium (DMEM, gibco, Thermo Fisher Scientific, MA, USA) containing 2 μg/mL TPCK (L-1-tosylamido-2-phenylethyl chloromethyl ketone)-treated trypsin, 100 U/mL penicillin, and 100 μg/mL streptomycin for 45 min at 37 °C. After adsorption, the supernatant was aspirated, and then cells were cultured with DMEM for the indicated time.

Human adenocarcinomic alveolar basal epithelial cells (A549), human embryonic kidney cells (HEK293T/293T), mouse embryo fibroblast cells (NIH/3T3), mouse Abelson murine leukemia virus transformed macrophages (RAW 264.7), mouse breast cancer cells (4T1) and Madin–Darby canine kidney cells (MDCK) cells were cultured in (DMEM gibco, Thermo Fisher Scientific, MA, USA) supplemented with 10% heat-inactivated fetal bovine serum (FBS, gibco, Thermo Fisher Scientific, MA, USA), 2 mM glutamine, 100 U/mL penicillin, and 100 μg/mL streptomycin. LncRNA ISR knockdown cell line was generated by infection of A549 cells with lentiviruses bearing short hairpin RNA (shRNA) targeting lncRNA ISR in pSIH-H1-GFP vector. A549 cells stably expressing lncRNA ISR or empty vector (EV) were generated by infecting with retroviruses encoding these genes in pNL-CMV-EGFP vector. RIG-I knockout cell line was generated using clustered regularly interspaced short palindromic repeats -associated protein 9 (CRISPR-Cas9) system as previously described [37].

### 4.2. Mice

Female 5- to 6-week-old *C57BL/6* mice were purchased from Vital River Laboratory Animal Center (Beijing, China). RIG-I KO mice on a *C57BL/6* background were obtained from Prof. Zhugang Wang (Shanghai Jiao Tong University School of Medicine, Shanghai, China). Mice were inoculated intranasally with 5 × 10^4^ pfu of IAV. Mice were euthanized 24 h after post infection, and then their organs were excised and collected for further analysis.

### 4.3. Ethics Statement

The animal care and use protocols were approved by the Research Ethics Committee of Institute of Microbiology, Chinese Academy of Sciences (Permit Number: SQIMCAS2018044, 14 September 2018–1 September 2021). All mouse experimental procedures were performed in accordance with the Regulations for the Administration of Affairs Concerning Experimental Animals approved by the State Council of People’s Republic of China.

### 4.4. Reverse-Transcription PCR and Quantitative PCR

Preparation of cDNA was performed using 2 μg of total RNA and Moloney murine leukemia virus (MMLV) reverse transcriptase (Promega, Madison, WI, USA) following RNA extraction. After cDNA synthesis, PCR and qPCR were conducted by rTaq DNA polymerase and SYBR Premix Ex Taq II, respectively (TaKaRa, Tokyo, Japan). The primers used in this study were mouse lncRNA ISR-forward (5′- CGTGCGACCAAAATCTCTCG-3′) and -reverse (5′- AGACAGTGTAGAACCCAGAGC-3′), human lncRNA ISR-forward (5′- GGCAAATGCATCCCTGCAAA -3′) and –reverse (5′- TCGCGGATTAGAGTGGTGTG -3′).

### 4.5. Hemagglutination Assay

Hemagglutination assay was modified from the method of Fazekas de St. Groth and Graham [38]. Briefly, 25 μL of PBS was added to each well of V-bottom 96-well microplates, followed by adding 25 μL of twofold diluted viral samples, and then 25 μL 1% chicken red blood cells were added to all wells. Samples were incubated at 37 °C for 20 min for reading the results.

### 4.6. Plaque Assay

MDCK cells were seeded in 12-well plates and incubated with serial dilutions of cell culture supernatants for 2 h. After incubation, cells were washed with PBS and then overlaid with DMEM containing 20% agarose and 0.1% TPCK-trypsin at 4 °C for 30 min. The plates were placed upside down at 37 °C for a further 72 h, followed by counting visible plaques for viral titer determination.

### 4.7. Statistical Analysis

Statistical comparison between two groups was analyzed by Student’s *t*-test. Data are represented as mean  ±  standard deviation (S.D.) with significant difference set at *p* < 0.05.

## Supplementary Materials

Supplementary materials can be found at https://www.mdpi.com/1422-0067/20/20/5118/s1.

## Abbreviations

A549	Human lung epithelial cells
BAHCC1	BAH domain and coiled-coil containing 1
CA04	A/California/04/2009
C57BL/6	C57 black 6
DMSO	Dimethyl sulfoxide
DMEM	Dulbecco’s Modified Eagle’s Medium
EV	Empty vector
GFP	Green fluorescent protein
H-lncRNA	Human Interferon-stimulated lncRNA
ISR	Interferon-stimulated lncRNA
hpi	Hours post infection
HSV	Herpes simplex virus
HA	Hemagglutination assay
HCV	Hepatitis C virus
ISR	Interferon-stimulated lncRNA
IFN-β	Interferon beta
IL-8	Interleukin-8
ISGs	Interferon-stimulated genes
ISG15	Interferon-stimulated gene 15
IRFs	Interferon regulatory factors
JAK-STAT	Janus Kinase- signal transducer and activator of transcription
M-lncRNA I	Mouse Interferon-stimulated lncRNA
MDA5	Anti-melanoma differentiation-associated gene 5
MIR155HG	miR-155 host gene
MOI	Multiplicity of infection
NIH/3T3	Mouse embryonic fibroblasts
NP	Nucleoprotein
NS1	Non-structural protein 1
NF-κB	Nuclear factor κ-light-chain-enhancer of activated B cells
NEAT1	Nuclear enriched abundant transcript 1
OAS2	Human 2’-5’-oligoadenylate synthetase 2
PAAN	PA-associated noncoding RNA
pfu	Plaque forming unit
PR8	A/Puerto Rico/8/1934
PRV	Pseudorabies virus
Poly (I:C)	Polyinosinic-polycytidylic acid
PRRs	Pattern recognition receptors
PBS	Phosphate-buffered saline
qRT-PCR	Quantitative Real Time-Polymerase Chain Reaction
RIG-I	Retinoic acid-inducible gene I
RAW264.7	Mouse Abelson murine leukemia virus transformed macrophages
RT-PCR	Reverse Transcriptase-Polymerase Chain Reaction
SFPQ	Splicing factor proline/glutamine-rich
SeV	Sendai virus
sh-Luc	Sh-luciferase
shRNA	Short hairpin RNAs
STAT3	Signal transducer and activator of transcription 3
TLR3	Toll-like receptor 3
TLR7	Toll-like receptor 7
TRIM25	Tripartite motif protein 25
WSN	A/WSN/1933(H1N1)
WT	Wild type mice
293T	HEK293T/Human embryonic kidney cells
4T1	Mouse breast cancer cells
