# Supplementary material for: Identification of an Interferon-Stimulated Long Noncoding RNA (LncRNA ISR) Involved in Regulation of Influenza A Virus Replication
Source: Int J Mol Sci. 2019 Oct 16;20(20):5118. doi: 10.3390/ijms20205118 (PMC6829313; doi:10.3390/ijms20205118)
Supplement: Supplementary file 1 [file ijms-20-05118-s001.zip › ijms-553112-SI.pdf]

Figure S1

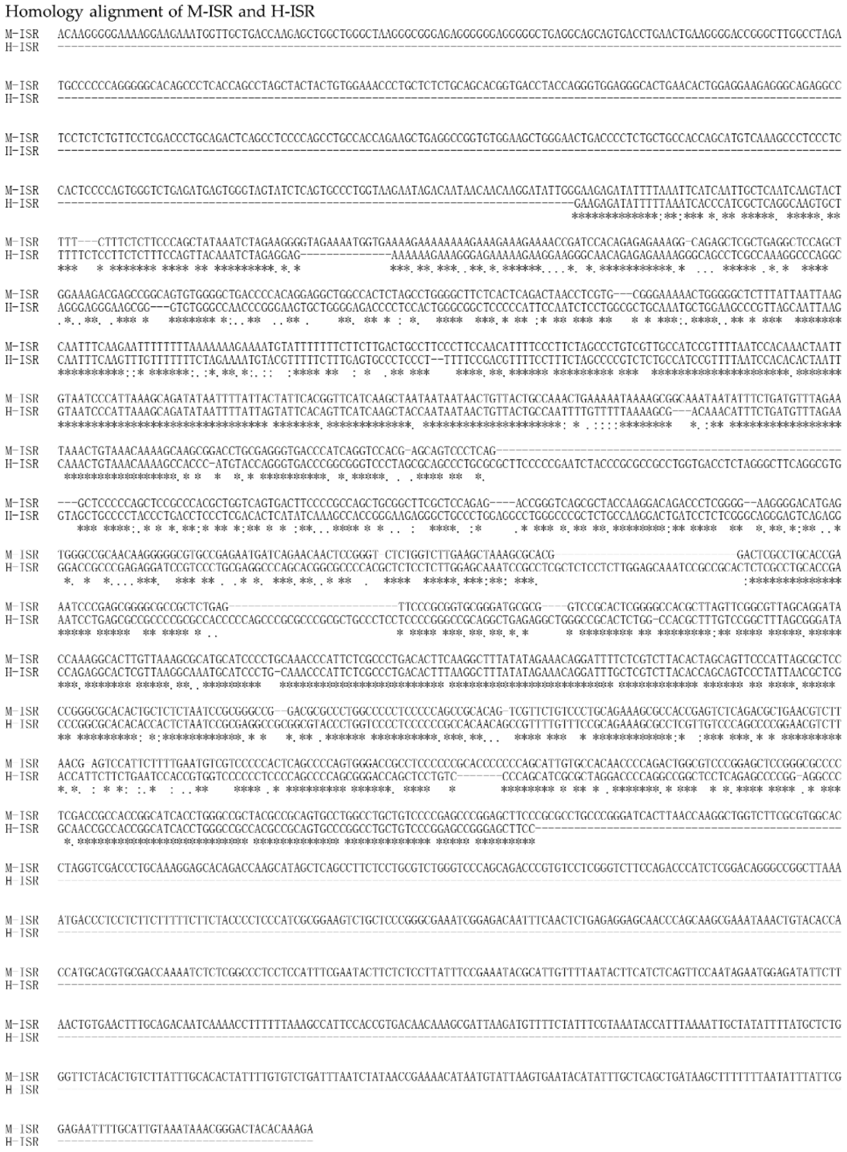

Figure S1. Homology alignment of M-ISR and H-ISR (https://www.genome.jp/tools-bin/clustalw).

Figure S2

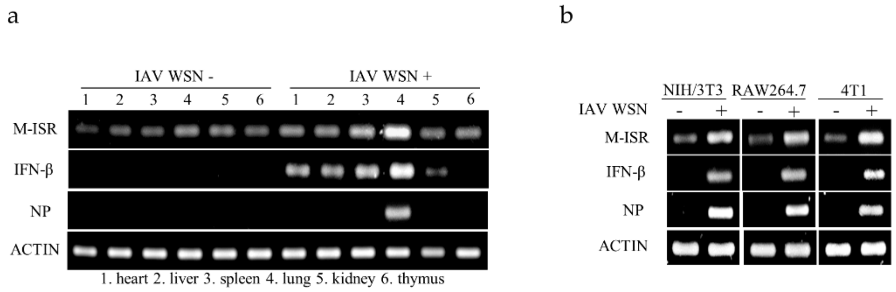

Figure S2. (a) The levels of lncRNA ISR and IFN-β expression in the organs of mice infected with or without IAV WSN for 24 hours were measured by RT-PCR. Lane 1-6: heart, liver, spleen, lung, kidney, and thymus; (b) The levels of lncRNA ISR and IFN-β in indicated mouse cell lines infected with or without IAV WSN (MOI=0.8) for 16 hours were examined by RT-PCR.
